# Draft Genome Sequences of Two *Salmonella enterica* Serotype Infantis Strains Isolated from a Captive Western Lowland Gorilla (*Gorilla gorilla gorilla*) and a Cohabitant Black and White Tegu (*Tupinambis merianae*) in Brazil

**DOI:** 10.1128/genomeA.01590-15

**Published:** 2016-01-21

**Authors:** Tatiane A. Paixão, Fernanda M. Coura, Marcelo C. C. Malta, Herlandes P. Tinoco, Angela T. Pessanha, Felipe L. Pereira, Carlos A. G. Leal, Marcos B. Heinemann, Henrique C. P. Figueiredo, Renato L. Santos

**Affiliations:** a Universidade Federal de Minas Gerais, Belo Horizonte, Brazil; b Fundação Zoo-Botânica de Belo Horizonte, Belo Horizonte, Brazil

## Abstract

The draft genome sequences of two *Salmonella enterica* serotype Infantis isolates are reported here. One of the strains was isolated from a western lowland gorilla (*Gorilla gorilla gorilla*) with colitis. The second strain was isolated from a reptile that inhabited the same premises. Whole-genome sequencing demonstrated that these isolates were not clonal.

## GENOME ANNOUNCEMENT

Salmonellosis is one of the most important foodborne infectious diseases worldwide. All Salmonella infections in warm-blooded animals and human patients are due to *Salmonella enterica* subsp. *enterica*, which includes >2,400 serotypes (1). Based on microbiological culture methods, reptiles traditionally have been considered reservoirs of *Salmonella* species (2). Here, we report the draft genome sequence of an *S. enterica* serotype Infantis strain (LPM-ST01) isolated from a 13-year-old female western lowland gorilla (*Gorilla gorilla gorilla*) kept in captivity in Belo Horizonte, Brazil, that developed fatal acute hemorrhagic colitis (3). A black and white tegu (*Tupinambis merianae*), a large South American reptile, was later found to inhabit the same premises as the infected gorilla. The tegu was captured and monitored for *Salmonella* species shedding by bacteriologic culture. One isolate from the tegu was identified by serotyping as *S*. Infantis (strain LPM-ST02), whose genome sequence is also reported here.

DNA from both strains was isolated from overnight culture with the Maxwell 16 tissue DNA purification kit using the Maxwell 16 system (both from Promega, USA). The genome was sequenced with the Ion Torrent PGM sequencing system (Life Technologies, USA) using a 400-bp-fragment library kit, according to the manufacturer’s recommendations. A total of 1,644,295 and 1,624,619 reads were obtained from LPM-ST01 and LPM-ST02, respectively, and were assembled using SPAdes 3.5 software (4), with the “—ion torrent—careful—k 21,33,55,77,97,127” parameters. These processes resulted in assemblies of 82 contigs with an *N*_50_ of 288,893 bp and 54 contigs with an *N*_50_ of 290,151 bp for LPM-ST01 and LPM-ST02, respectively.

This genomic analysis clearly demonstrates that these two *S*. Infantis isolates were not clonal, which is quite relevant information for the control and prevention of salmonellosis from a conservational veterinary medicine perspective. Although reptiles have been considered reservoirs of *Salmonella* spp., this assumption was largely based on bacteriologic culture. More recently, high-throughput sequencing methods demonstrated distinct populations associated with reptiles and mammalian hosts, which does not support the traditional assumption that reptiles are reservoirs of *Salmonella* species (2). To the best of our knowledge, there is only one previously reported genome sequence of *S*. Infantis (4), so this report will contribute to the efforts of future comparative genomic studies.

### Nucleotide sequence accession numbers.

This whole-genome shotgun project has been deposited in DDBJ/ENA/GenBank under the accession numbers LMZA00000000 and LMZB00000000 for strains LPM-ST01 and LPM-ST02, respectively. The versions described in this paper are the first versions, LMZA01000000 and LMZB010000000.
